# Prevalence and predictors of problematic alcohol use among Hungarian physicians: associations with specialty, burnout dimensions, anxiety and perceived stress

**DOI:** 10.1192/j.eurpsy.2026.10208

**Published:** 2026-07-31

**Authors:** O. Csabai, T. T. Simonyi, K. Kostyál, I. K. Pribék, K. M. Hegedűs, A. Kukla, D. Varga, B. Paksi, Z. Demetrovics, P. Z. Álmos

**Affiliations:** 1 Department of Psychiatry, University of Szeged, Szeged; 2 Institute of Education, ELTE Eötvös Loránd University, Budapest, Hungary; 3 Institute for Mental Health and Wellbeing, College of Education, Psychology and Social Work, Flinders University, Bedford Park, Australia; 4 Institute of Psychology, ELTE Eötvös Loránd University, Budapest, Hungary; 5 Centre of Excellence in Responsible Gaming, University of Gibraltar, Gibraltar, Gibraltar

## Abstract

**Introduction:**

Physicians are vulnerable to burnout, depression, and anxiety. Occupational stress may predispose them to problematic alcohol use, affecting health, well-being, and patient care.

**Objectives:**

This study investigates the prevalence of problematic alcohol use among physicians in Hungary and examines its associations with burnout, perceived stress, anxiety, and medical specialty.

**Methods:**

Between November 2024 and June 2025, a nationwide cross-sectional study was conducted using an anonymous online survey with randomly selected participants. A total of 8 000 physicians from across Hungary were invited to participate. The study included specialists, specialist trainees, residents, dentists and psychologists. The questionnaire included items on demographic characteristics, the Alcohol Use Disorders Identification Test (AUDIT), the State-Trait Anxiety Inventory (STAI-5), Maslach Burnout Inventory (MBI) and the Perceived Stress Scale (PSS).

**Results:**

A total of 2826 participants initiated the survey, of which 1380 (17.2%) were fully completed. Among the respondents, 439 (31.8%) were male and 941 (68.2%) female physicians. Participants’ ages ranged from 24 to 88 years, with a mean age of 50.32 years (SD = 15.36). Based on the Alcohol Use Disorders Identification Test (AUDIT; ≥8) (*n* = 1374, missing = 6), 1252 physicians (91.1%) were classified as non-problematic drinkers, while the prevalence of problematic alcohol use was 122 (8.9%). Harmful alcohol use (>15) was identified in 9 participants (0.7%), and probable alcohol dependence (≥20) in 2 participants (0.1%). Across medical specialties, the highest rates of problematic alcohol use were observed in surgery (20.35%), internal medicine (8.37%) and dentistry (7.3%). Binary logistic regression analyses of the Maslach Burnout Inventory subscales indicated that emotional exhaustion (*χ²*(1) = 24.43, *p* < 0.001), depersonalization (*χ²*(1) = 41.61, *p* < 0.001) and reduced personal accomplishment (*χ²*(1) = 9.51, *p* = 0.002) significantly predicted problematic alcohol use. Furthermore, weak but significant positive correlations were found between problematic alcohol use and state anxiety (*r*_s_(1367) = 0.149, *p* < 0.001), trait anxiety (*r_s_*(1370) = 0.122, *p* < 0.001) and perceived stress (*r_s_*(1355) = 0.101, *p* < 0.001).

**Conclusions:**

Problematic alcohol use among physicians is important but understudied. In our sample, prevalence was 8.9%, while harmful use (0.7%) and probable dependence (0.1%) were rarer. Highest rates were seen in surgical, internal medicine, and dental fields. Burnout dimensions — emotional exhaustion, depersonalization, and low personal accomplishment — significantly predicted problematic alcohol use. Furthermore, our findings raise the possibility of an association between state and trait anxiety, as well as perceived stress, and problematic alcohol consumption.

**Disclosure of Interest:**

O. Csabai Grant / Research support from: This abstract is financially supported by Hetényi Géza grant (number: 5S 764 (A202)) from SZAOK Faculty Research Fund., T. Simonyi: None Declared, K. Kostyál: None Declared, I. Pribék: None Declared, K. Hegedűs: None Declared, A. Kukla: None Declared, D. Varga: None Declared, B. Paksi: None Declared, Z. Demetrovics: None Declared, P. Álmos: None Declared

